# Neuromuscular Activity of *Micrurus laticollaris* (Squamata: Elapidae) Venom *in Vitro*

**DOI:** 10.3390/toxins6010359

**Published:** 2014-01-17

**Authors:** Alejandro Carbajal-Saucedo, Rafael Stuani Floriano, Cháriston André Dal Belo, Alejandro Olvera-Rodríguez, Alejandro Alagón, Léa Rodrigues-Simioni

**Affiliations:** 1 Instituto de Biotecnología, Universidad Nacional Autónoma de México, Av. Universidad 2001, Col. Chamilpa, Cuernavaca, Morelos, CP. 62210, Mexico; E-Mails: micropechis@yahoo.com.mx (A.C.-S.); aolvera@ibt.unam.mx (A.O.-R.); alagon@ibt.unam.mx (A.A.); 2 Department of Pharmacology, Faculty of Medical Sciences, State University of Campinas (UNICAMP), Tessália Vieira Camargo St., 126, Campinas, SP 13083-887, Brazil; E-Mail: floriano_rs@yahoo.com; 3 Interdisciplinar Center for Biotechnology (CIPBiotec), Campus São Gabriel, Federal University of Pampa (UNIPAMPA), Antônio Trilha Av., 1847, São Gabriel, RS 97300-000, Brazil; E-Mail: charistondb@gmail.com

**Keywords:** snake venom, *Micrurus laticollaris*, presynaptic action, postsynaptic action, neuromuscular preparations

## Abstract

In this work, we have examined the neuromuscular activity of *Micrurus laticollaris* (Mexican coral snake) venom (MLV) in vertebrate isolated nerve-muscle preparations. In chick biventer cervicis preparations, the MLV induced an irreversible concentration- and time-dependent (1–30 µg/mL) neuromuscular blockade, with 50% blockade occurring between 8 and 30 min. Muscle contractures evoked by exogenous acetylcholine were completely abolished by MLV, whereas those of KCl were also significantly altered (86% ± 11%, 53% ± 11%, 89% ± 5% and 89% ± 7% for one, three, 10 and 30 µg of venom/mL, respectively; *n* = 4; *p* < 0.05). In mouse phrenic nerve-diaphragm preparations, MLV (1–10 µg/mL) promoted a slight increase in the amplitude of twitch-tension (3 µg/mL), followed by neuromuscular blockade (*n* = 4); the highest concentration caused complete inhibition of the twitches (time for 50% blockade = 26 ± 3 min), without exhibiting a previous neuromuscular facilitation. The venom (3 µg/mL) induced a biphasic modulation in the frequency of miniature end-plate potentials (MEPPs)/min, causing a significant increase after 15 min, followed by a decrease after 60 min (from 17 ± 1.4 (basal) to 28 ± 2.5 (*t*_15_) and 12 ± 2 (*t*_60_)). The membrane resting potential of mouse diaphragm preparations pre-exposed or not to d-tubocurarine (5 µg/mL) was also significantly less negative with MLV (10 µg/mL). Together, these results indicate that *M. laticollaris* venom induces neuromuscular blockade by a combination of pre- and post-synaptic activities.

## 1. Introduction

Coral snakes (formally included in the genera, *Micrurus*, *Leptomicrurus* and *Micruroides*) constitute a taxonomic assemblage of more than 120 species and subspecies [1,2,3] distributed from the southern United States to southern South America, achieving their greatest abundance of species in lower latitudes [4], and they are, together with the pelagic sea snake, *Pelamis platurus*, the American representatives of the family, Elapidae [5,6]. Their venoms contain low-molecular mass neurotoxins of the three-finger type, which block the neuromuscular junction by binding with high affinity to the cholinergic receptor at the neuromuscular junction, promoting a paralytic effect [7,8,9,10]. In addition, *Micrurus* venoms contain phospholipase A_2_, which can induce myotoxicity [11,12,13,14], hemorrhage [15], inflammation [16] and presynaptic neurotoxicity [17,18].

*Micrurus laticollaris* is one of the most important snakes of medical importance in Mexico [19]. Recently, a short-chain alpha-neurotoxin has been isolated from *M. laticollaris* venom, which appears to be determinant in the whole envenomation [20]. However, data related to the interaction of *M. laticollaris* crude venom at the neuromuscular junction are scarce and become a valuable source of information to improve the clinical treatment of snakebite accidents. In addition, in spite of its obvious clinical interest, the study of the whole venom uncovers the presence of other toxins that are no less important in terms of potential medical applications. In this work, we investigated the neuromuscular activity of the crude venom from the Mexican coral snake, *M. laticollaris*.

## 2. Results and Discussion

**Figure 1:**
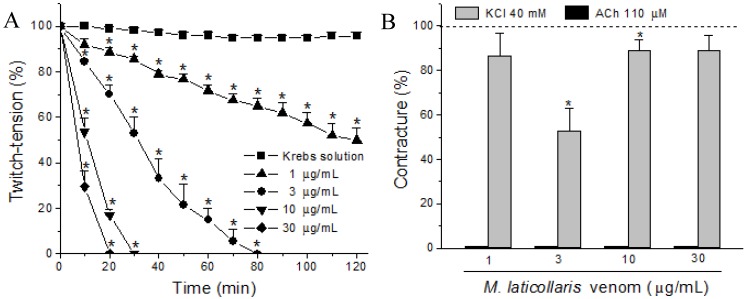
Neuromuscular blockade caused by *M. laticollaris* venom (one, three, 10 and 30 µg/mL) in biventer cervicis (BC) preparations. (**A**) Graphic of the concentration- and time-dependent blockade; (**B**) Columns representing muscle contractures to exogenous ACh (110 µM) and KCl (40 mM) after 120 min of incubation or complete neuromuscular blockade are expressed as a percentage of the responses observed in control preparations (Krebs solution alone). The points in **A** and the columns in **B** are the mean ± SEM (*n* = 4); *p < 0.05 compared to control preparations in both A and B panels.

*Micrurus laticollaris* venom (1–30 µg/mL) caused irreversible time- and concentration-dependent neuromuscular blockade in indirectly stimulated biventer cervicis (BC) preparations, with complete blockade occurring from 20 min at the highest concentration (30 µg/mL) (Figure 1A). The times required for 50% blockade were 36 ± 4.6, 11 ± 0.9 and 8 ± 0.6 min for three, 10 and 30 µg venom/mL, respectively. All tested venom concentrations completely inhibited the contractures to exogenous ACh (110 µM) after neuromuscular blockade, whereas contractures to KCl (40 mM) had a minor change (86 ± 11, 53 ± 11, 89 ± 5 and 89 ± 7 for 1, 3, 10 and 30 µg of venom/mL, respectively, expressed as a percentage of the control, considered as 100%; *n* = 4 each) (Figure 1B). Figure 3A shows a representative record of the neuromuscular blockade caused by a venom concentration of 10 µg/mL and contracture responses to ACh and KCl before and after venom treatment. The decrease of the twitch tension in avian neuromuscular preparations caused by Elapidic venoms has been previously shown [13,21,22,23,24,25]. At these preparations, the muscle-type nicotinic receptors are distributed alongside the muscle fiber characterizing the multiple neuromuscular junctions [26]. Frequently, in multi-innervated nerve-muscle preparations treated with Elapidic venoms, the decrease of the amplitude of muscle twitches is accompanied by the attenuation or complete blockade of the muscle contracture induced by exogenous ACh. In these preparations, the inhibition of both twitch-tension and muscle contracture response to exogenous ACh caused by Elapidic venoms is known to be due the presence of alpha-neurotoxins [27,28,29]. These toxins act specifically on neuronal and muscle-type nicotinic receptors, preventing the binding of Ach, which inhibits the sodium influx and, consequently, the membrane depolarization [20,28]. Indeed, a novel short-chain alpha neurotoxin was identified from *Micrurus laticollaris* venom, which is able to inhibit the biding of ACh at cloned nicotinic receptors expressed at *Xenopus laevae* [20].

**Figure 2:**
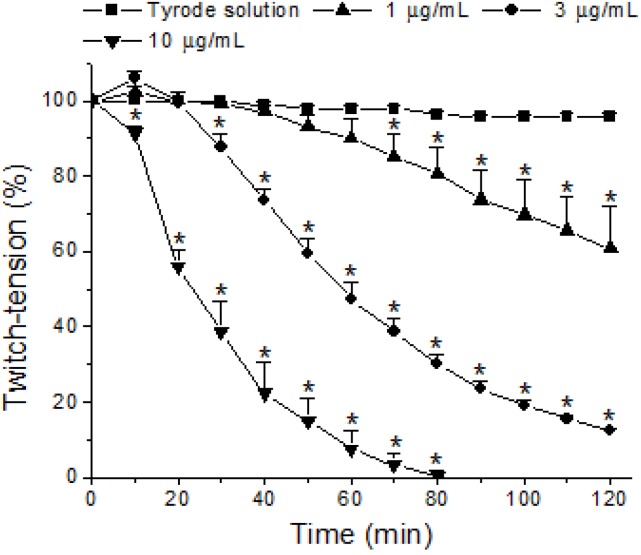
Concentration- and time-dependent neuromuscular blockade caused by *M. laticollaris* venom (one, three and 10 µg/mL) in phrenic-nerve diaphragm (PND) preparations. Note that a 3 µg/mL venom concentration caused a minor neuromuscular facilitation after 10 min of incubation. The points are the mean ± SEM (*n* = 4); * *p* < 0.05 compared to control preparations.

**Figure 3:**
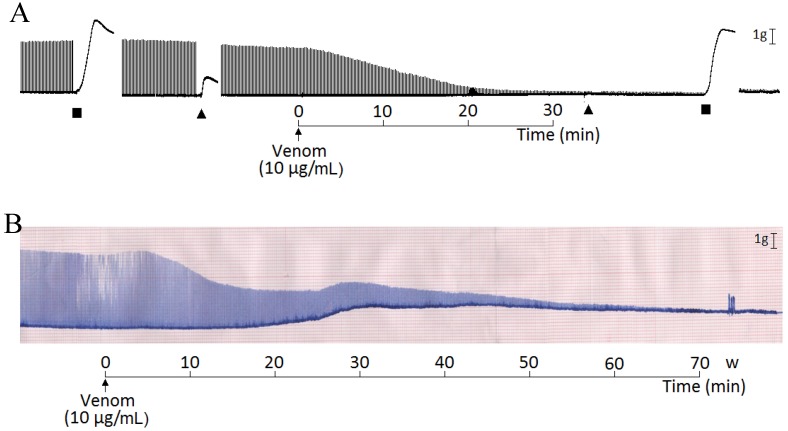
Neuromuscular blockade caused by *M. laticollaris* venom in BC and PND preparations. (**A**) Representative recordings showing the blockade caused by *M. laticollaris* venom (10 µg/mL) in a BC preparation indirectly stimulated at 37 °C. Note that responses to exogenous ACh (▲, 110 µM) were completely abolished by venom, whereas those to KCl (■, 40 mM) had a minor decrease; (**B**) Representative recordings showing the blockade caused by *M. laticollaris* venom (10 µg/mL) in a PND preparation indirectly stimulated at 37 °C. This venom concentration caused contracture in the baseline from 20 min of incubation.

In mouse phrenic-nerve diaphragm (PND) preparations, the *M. laticollaris* venom (one, three and 10 µg/mL) also produced irreversible time- and concentration-dependent blockade, although less active than that of biventer cervicis (BC) preparations, with complete neuromuscular blockade occurring just at the highest concentration (10 µg/mL; time for 50% blockade: 26 ± 3 min) after 80 min of incubation, whereas 39% ± 11% and 88% ± 1% blockade for one and 3 µg/mL, respectively, were observed after 120 min. (Figure 2). Figure 3B, the PND preparation, shows a representative record of the neuromuscular blockade caused by the venom (10 µg/mL) preceded by a marked increase in the baseline (contracture). The difference in sensitivity of the chick biventer cervicis and mouse diaphragm preparations to *M. laticollaris* venom is a fact. The explanation must rely not only the species selectivity of animal toxins [30,31], but also on the anatomical differences between mouse diaphragm (single innervated fiber, en plaque endings) and chick biventer end-plates (multiple innervated fiber, en grape endings) [32]. Furthermore, the existence of two populations of nicotinic receptors in BC preparations is well-known [33,34], *i.e*., one population that is extrajunctional and responds well to exogenous Ach, but is rapidly blocked by venom neurotoxins, and another located in the motor end-plate that responds to nerve stimulation and is less susceptible to rapid blockade by neurotoxins (possibly because of difficulties related to toxin diffusion into the synaptic cleft). This also agrees with studies for other neurotoxins that discriminate between these two populations [35,36] (Chang *et al.*, 1973; Chang and Su, 1975). Chemical differences between junctional and extrajunctional receptors obviously could also be a clue to the molecular mechanisms involved in generating and maintaining these differences. Studies using chimeric subunits and site-direct mutagenesis have demonstrated the importance of amino acids located within loops D, E and F in determining selectivity for nicotinic agonists and antagonists [37,38,39,40]. In addition, the differences in maturity of the nicotinic receptors present in both species must complement a reasonable explanation. To date, the neuromuscular nAChR is a pentamer comprised of two α1 subunits, one β1 subunit, one δ subunit and either a γ or an ε subunit. In mammals, the γ subunit (fetal subtype) is replaced by the ε subunit (adult subtype) during development. In mice, the ε subunit is known to replace the γ subunit during the first 14 days postnatal [41]. Normally, both presynaptic and postsynaptic effects can be observed in murine nerve-muscle preparations assayed with Elapidic venoms [22,42]. The presence of PLA_2_-neurotoxins in these venoms induces a triphasic effect, *i.e*., the increase of the muscle twitch-tension amplitude before the irreversible neuromuscular blockade [21], as it was also observed in PND preparations treated with 3 µg of *M. laticollaris* venom/mL (see Figure 2). In addition, the initial neuromuscular facilitation caused by *Micrurus* venoms could be a result of an increase in neurotransmitter release [42] that can be partly due to a modulation of miniature end-plate potential (MEPP) parameters [21,43].

**Figure 4:**
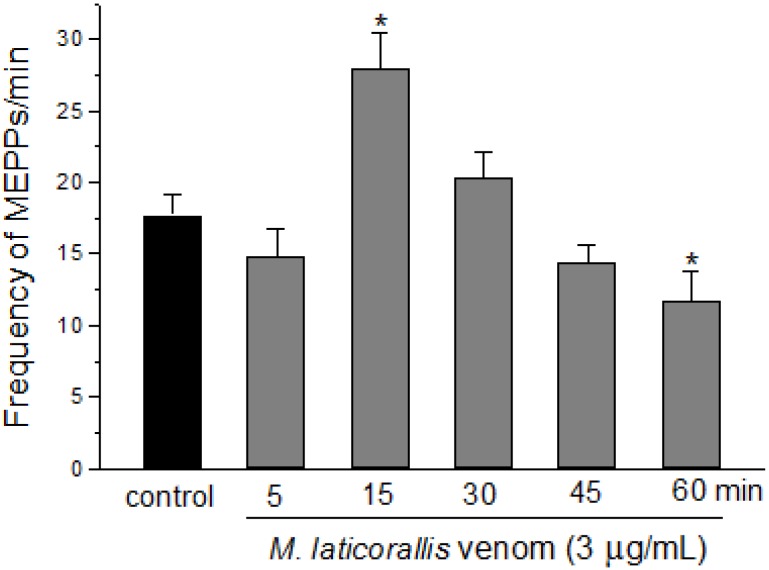
Miniature end-plate potential (MEPP) measurements in PND preparations treated with *M. laticollaris* venom. The columns are the mean ± SEM of four muscles in which three different end-plates were measured per interval; * *p* < 0.05 compared to *t*_0_ (control) values.

Indeed, *M. laticollaris* venom (3 µg/mL) induced a significant increase in the frequency of miniature end-plate potentials (MEPPs/min) in mouse diaphragm after 15 min and then a decrease after 60 min (from 17 ± 1.4 (basal) to 28 ± 2.5 (*t*_15_) and 12 ± 2 (*t*_60_); *n* = 4) (Figure 4), with no important alteration in the MEPPs’ amplitude (mV: 1.2 ± 0.02, 1.4 ± 0.02, 1.3 ± 0.02, 1.2 ± 0.02, 1.1 ± 0.02 and 1.2 ± 0.02 for *t*_0_ (basal), *t*_5_, *t*_15_, *t*_30_, *t*_45_ and *t*_60_ min, respectively, *n* = 4). As discussed above, this effect could contribute to an increase of neurotransmitter release before the onset of neuromuscular blockade. This conclusion also exposes the presence of presynaptic neurotoxins in *M. laticollaris* venom, which are probably related to PLA_2_-neurotoxin actions [21,22,42,43].

**Figure 5:**
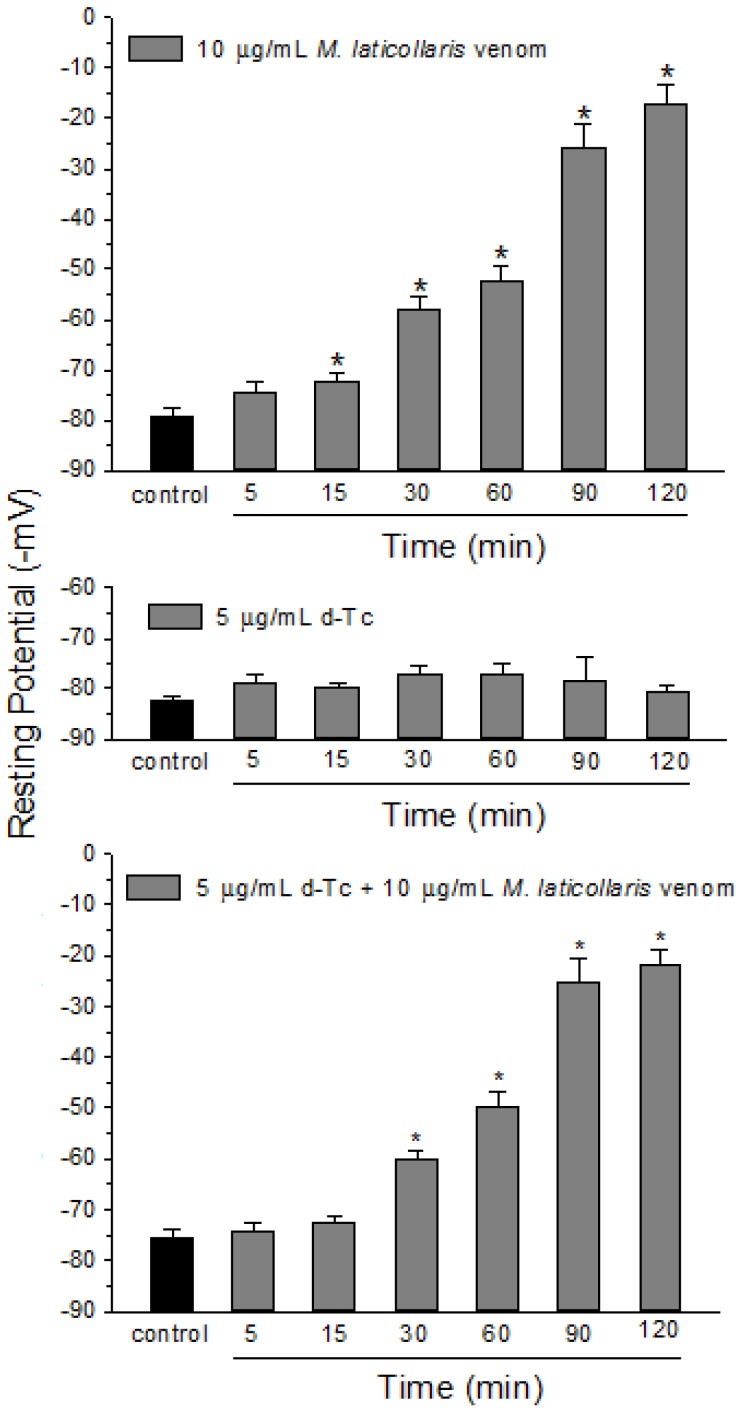
Membrane resting potential measurements in curarized and uncurarized PND preparations treated with *M. laticollaris* venom. (**A**) The depolarizing effect caused by venom (10 µg/mL) in uncurarized preparations; (**B**) The lack of depolarization by d-tubocurarine (d-Tc, 5 µg/mL) after 120 min of incubation; (**C**) The depolarizing effect caused by venom (10 µg/mL) in preparations previously treated with d-Tc (5 µg/mL) for 10 min. Note that pretreatment with d-Tc did not prevent the depolarizing effect caused by venom. The columns are the mean ± SEM of four muscles in which six different end-plates were measured per interval; * *p* < 0.05 compared to *t*_0_ (control) values in all panels.

It was also observed that the venom (10 µg/mL) caused a significant membrane depolarization from 15 min of incubation in PND preparations (from −79 ± 1.6 mV (*t*_0_) to −17 ± 3.7 mV (*t*_120_); *n* = 4) (Figure 5, higher panel). Figure 5 (intermediate panel) is a positive control experiment that shows the lack of depolarization in preparations incubated with d-tubocurarine (d-Tc, 5 µg/mL) for 120 min (from −82 ± 1 mV (*t*_0_) to −81 ± 1.3 mV (*t*_120_); *n* = 4). However, preparations pretreated with d-Tc (5 µg/mL) for 10 min and then exposed to venom (10 µg/mL) also exhibited important membrane depolarization from 30 min of venom incubation (from−75 ± 1.7 mV (*t*_0_) to −22 ± 3 mV (*t*_120_); *n* = 4) (Figure 5-lower panel). Myotoxicity is a common phenomenon related to the activity of *Micrurus* venoms in nerve-muscle preparations [38,40,41]. PLA_2_-myotoxins present in *Micrurus* venoms are responsible for morphological alterations in skeletal muscle [21,38,42]. In addition, the activity of PLA_2_-myotoxins in nerve-muscle preparations normally induces a decrease in the resting membrane potential [21,43,44,45,46]. We suggest that the membrane depolarization caused by *M. laticollaris* as observed here, apart from alteration of the baseline and changes in the contracture to KCl in the twitch-tension experiments, can also be related to the presence of PLA_2_-myotoxins in the composition of this venom.

## 3. Experimental Section

### 3.1. Venom and Animals

*Micrurus laticollaris* venom (Mexican coral snake) was provided, in a lyophilized form, by Instituto de Biotecnología of the Universidad Nacional Autónoma de México (Cuernavaca, Morelos, Mexico). When required, the venom was dissolved in Krebs or Tyrode solution prior to use. Male Swiss mice (25–30 g) obtained from the Multidisciplinary Center for Biological Investigation (CEMIB/Unicamp) were housed 10/cage at 23 °C on a 12 h light/dark cycle with lights on at 6 a.m. Male HY-LINE chicks (4–8 days old) were supplied by Globo Aves Agricola Ltda. (Campinas, SP, Brazil) and were housed in metal cages with a sawdust substrate. The mice and chicks had free access to food and water.

### 3.2. Twitch-Tension Experiments

Chick biventer cervicis nerve-muscle preparations (BC) were mounted (resting tension: 1 g) in Krebs solution (composition, in mM: NaCl 119, KCl 4.7, CaCl_2_ 1.9, KH_2_PO_4_ 1.2, MgSO_4_ 1.2, NaHCO_3_ 25 and glucose 11.7, pH 7.5 at 37 °C, after equilibration with 95% O_2_/5% CO_2_) and allowed to stabilize for 20 min prior to use, as described elsewhere [47,48]. Field stimulation (0.1 Hz, 0.2 ms) were delivered from an LE 12406 TC stimulator (Panlab, Spain), and the muscle twitches were recorded using a TRI201AD force displacement transducer coupled to a Quad Bridge Amp and LabChart 6.0 software (all from ADInstruments Pty Ltd., Bella Vista, Australia). Muscle responses to exogenous acetylcholine (ACh, 110 µM) and KCl (40 mM) were obtained before and after incubation with venom (1–30 µg/mL) to screen for postsynaptic neurotoxicity and myotoxicity [49].

Mouse phrenic nerve-diaphragm preparations (PND) were mounted in Tyrode solution (composition, in mM: NaCl 137, KCl 2.7, CaCl_2_ 1.8, MgCl_2_ 0.49, NaH_2_PO_4_ 0.42, NaHCO_3_ 11.9 and glucose 11.1, pH 7.0 at 37 °C, after equilibration with 95% O_2_/5% CO_2_), as described elsewhere [48,50]. Supramaximal stimuli (0.1 Hz and 0.2 ms) were delivered to the nerve from a Grass S88 stimulator (Grass Instrument Co., Quincy, MA, USA), and the muscle twitches were recorded using a Load Cell BG-25 g force displacement transducer coupled to a Gould RS 3400 recorder (both from Gould Inc., Cleveland, OH, USA). After stabilization for 20 min, the preparations were incubated with different venom concentrations (1, 3 and 10 µg/mL) for 120 min or complete neuromuscular blockade and the changes in twitch-tension recorded.

### 3.3. Intracellular Recordings

The effect of the venom on the frequency of miniature end-plate potentials (MEPPs) and membrane resting potential (RP) was recorded using mouse hemidiaphragm muscle mounted in a Lucite chamber containing Tyrode solution (composition shown above), as described previously elsewhere [24,48]. The MEPPs frequency was measured in multiple neuromuscular junctions at various intervals from control (*t*_0_) and venom-treated preparations (*t*_5_–*t_60_*). For RP measurements, different regions of the muscle were monitored at various intervals from control (*t*_0_) and venom-treated preparations (*t*_5_−*t*_120_ for RP); some preparations were pre-incubated with d-tubocurarine (d-Tc, 5 µg/mL), a non-depolarizing compound, to verify the venom activity on nicotinic receptors and/or the muscle membrane.

### 3.4. Statistical Analysis

Changes in the twitch-tension responses of BC and PND preparations and the membrane resting potential of PND preparations were expressed as a percentage relative to baseline (time zero) values. The results were expressed as the mean ± SEM, and statistical comparisons were done using the Student’s *t*-test or ANOVA, followed by the Tukey test, with *p* < 0.05 indicating significance. All data analyses were done using Microcal Origin software (Microcal Software Inc., Northampton, MA, USA).

## 4. Conclusions

Based on these results, we conclude that *Micrurus laticollaris* venom causes neuromuscular blockade in avian and mammalian preparations *in vitro* essentially by a combined postsynaptic action between nicotinic receptor blockade and muscle membrane resting potential depolarization, since contracture responses to exogenous ACh in BC preparations was completely inhibited by all venom concentrations tested, and there was a significant depolarization of the muscle fiber membrane caused by venom in PND preparations. The venom-induced blockade was preceded by a presynaptic effect, which initially facilitates neurotransmitter release, as shown by the increase in twitch-tension amplitude and the frequency of MEPPs in PND preparations, suggesting also the presence of β-neurotoxins in the crude venom.
